# A Collective Intelligence Strategy for Evaluating and Advancing Nurse Autonomy in Primary Care

**DOI:** 10.3390/healthcare13121403

**Published:** 2025-06-12

**Authors:** Alba Brugués Brugués, Jèssica Morillas Vázquez, Enric Mateo Viladomat, Glòria Jodar Solà, Michelle Catta-Preta, Alex Trejo Omeñaca, Jan Ferrer i Picó, Josep Maria Monguet i Fierro

**Affiliations:** 1 Associació d’Infermeria Familiar i Comunitària de Catalunya, Carrer Pujades 350, 08019 Barcelona, Catalonia, Spain; 2 CatSalut, Edifici Olimpia, Travassera de les Corts 131-159, 08028 Barcelona, Catalonia, Spain; 3 Institut Català de la Salut, Gran Via de les Corts Catalanes 587, 08007 Barcelona, Catalonia, Spain; 4 Consorci Castelldefels Agents de Salut (CASAP), Av. Ciutat de Màlaga 18-20, 08860 Castelldefels, Catalonia, Spain; 5 Innex Labs, Carrer de Tarragona 10, 08800 Vilanova i la Geltrú, Catalonia, Spainjosep@innex.io (J.M.M.i.F.); 6 Departament d’Enginyeria Gràfica i Disseny, Escola Politècnica Superior d’Enginyeria de Vilanova i la Geltrú (EPSEVG), Universitat Politècnica de Catalunya, Avinguda de Víctor Balaguer 1, 08800 Vilanova i la Geltrú, Catalonia, Spain; 7 Departament d’Enginyeria Gràfica i Disseny, Escola Tècnica Superior d’Enginyeria Industrial de Barcelona (ETSEIB), Universitat Politècnica de Catalunya, Avinguda Diagonal 647, 08028 Barcelona, Catalonia, Spain

**Keywords:** primary care, nurse demand management, nurse autonomy, collective intelligence, participation, evaluation metrics

## Abstract

Background: European health systems are shifting toward more proactive, person-centered models, thereby highlighting the need to strengthen nurses’ clinical leadership in primary care. Nurse demand management (NDM) has emerged as an innovative practice which allows nurses to autonomously and comprehensively respond to a population’s health needs. However, knowledge on its implementation varies widely, often being intuitive, partly due to the absence of standardized evaluation tools. The xGID instrument aims to measure the degree of NDM adoption in primary care teams (PCTs), activating collective intelligence mechanisms to foster shared diagnosis, organizational reflection, and the generation of targeted recommendations. Methods: We designed and implemented xGID in 47 PCTs in Catalonia, involving 1474 healthcare professionals. Data were collected through structured surveys assessing key dimensions of NDM adoption, including professional autonomy, teamwork, continuity, and accessibility. Results: Overall adoption of NDM was high, with a mean score of 7.6 out of 10. Notable differences emerged between professional groups and practice areas. Nurses tended to be more critical of teamwork, longitudinal care, and accessibility, reflecting the central yet high-pressure role they play in NDM. High-scoring dimensions included professional autonomy and the capacity to act across multiple domains, whereas weaker areas pointed to systemic organizational challenges. Conclusions: The preliminary findings indicate that a standardized tool for NDM evaluation is a cornerstone for identifying contextual barriers and guiding the transformation of care models. Its participatory and strategic approach offers novel pathways to embed data-driven decision-making into daily clinical practice, consolidating NDM as a key pillar of future primary care.

## 1. Introduction

The increasing complexity of health needs—driven by population ageing, multimorbidity, and the growing burden of chronic diseases—has exposed the limitations of care models centred exclusively on the physician. In response, health systems throughout Europe are moving toward more integrated, proactive, and person-centred approaches. Many countries are reorientating primary care delivery to multidisciplinary teams as a strategy to improve quality and access [1,2,3]. Within this transformation, the development of new professional roles—especially in the field of nursing—has become a key driver of change [3,4]. Skill-mix strategies, which involve the redistribution of roles, the emergence of new professional profiles, and shared leadership, have been widely examined for their capacity to enhance efficiency and responsiveness in primary care. There is growing evidence that transferring clinical responsibilities to nurses is associated with positive outcomes regarding access, clinical effectiveness, cost efficiency, and patient satisfaction [5,6]. For example, since the 1980s, Spain has prioritized community-based, multi-profile primary care teams, composed of family physicians, nurses, and other professionals working together to develop joint care plans and share clinical responsibilities [7].

In Catalonia, this paradigm shift has taken shape through the development of the nurse demand management (NDM) model. Developed in 2009 amid primary care reforms, NDM was introduced to reorganize the flow of same-day patient demand and improve access to care [8,9]. The NDM model is a formal programme led by primary care nurses to allow them to autonomously attend to patients with acute minor illnesses under defined clinical protocols. Nowadays, NDM refers to far more than same-day flows and includes the autonomous clinical response of nurses to patients’ needs, founded on holistic assessment, clinical judgement, and the creation of a care plan aligned with nursing processes. This model positions nurses not only to address “what the individual has” but also “what the individual needs”, reflecting a salutogenic and biopsychosocial perspective. As the first nurse-led clinical self-regulation process in Catalonia, NDM has shown benefits in terms of accessibility, continuity of care, and the resolution of common health concerns [10].

NDM has shown promising clinical outcomes. A large-scale evaluation of the expanded nurse-led urgent care initiative (2009–2010) reported that nurses successfully resolved most same-day consultations for minor ailments, with resolution rates of approximately 62% in adults and 76% in pediatric cases [9]. Only a small proportion required follow-up with a physician or return visits, with 7-day revisit rates of 4.0% for adults and 2.4% for children, indicating that nurse-led management was both safe and effective [9]. The study concluded that large-scale deployment of nurses for minor acute conditions is feasible and yields high resolution rates with minimal relapse, validating NDM as an effective demand-management strategy.

High patient satisfaction with nurse-led acute care has also been reported in related primary care settings outside the specific Catalonian context [11], suggesting strong patient acceptance when care quality is maintained. By 2014, approximately 70% of Catalonia’s primary care providers had adopted NDM, reflecting rapid regional diffusion [12]. This widespread uptake occurred despite initial barriers such as unclear legal authority for nurse prescribing, variability in training, and the need for new clinical protocols [12]. These challenges were addressed through targeted training programmes, professional consensus-building, and policy measures, including regulatory updates and incentive alignment [12]. Nonetheless, implementation remains uneven, highlighting the need for systematic tools to evaluate actual uptake and identify persistent gaps—an area previously lacking structured assessment [13].

In this context, digital tools such as xGID (“x” for experience, and GID referencing the Catalan acronym for NDM) have emerged as promising catalysts for transformation. xGID is a participatory digital system designed to measure and visualize NDM adoption within primary care teams from both practice and managerial perspectives. It serves not only as a monitoring tool but also as a collective intelligence mechanism, fostering internal dialogue and organizational learning through shared indicators and structured reflection [14]. Conceptually, it builds on the Nursing Role Effectiveness Model [15], integrating structural, process, and outcome dimensions to evaluate nurse-sensitive performance. Clinical data are intentionally excluded, as the focus is on implementation rather than outcomes, which have already been demonstrated elsewhere [9].

This article presents the design, implementation, and preliminary results of xGID in a representative sample of 47 primary care teams in Catalonia. The aim is to assess the level of NDM implementation and explore the tool’s potential to strengthen professional autonomy and organizational capacity within European primary care systems.

The overall objective of this project is to evaluate the degree of adoption of NDM in primary care teams (PCTs) in Catalonia. The objectives of this paper are divided as follows:

1. Quantify the level of NDM implementation at each participating centre, based on the results obtained from the 19 assessment items included in the xGID tool.
2. Analyze the degree of internal consensus across different professional profiles (nurses, physicians, administrative staff, and others) regarding NDM application, identifying both divergences and potential synergies.
3. Identify the main facilitators and barriers to the effective implementation of NDM, encompassing organizational, educational, cultural, and clinical leadership aspects.
4. Explore the influence of individual variables—such as professional profile—on the perception and evaluation of NDM.
5. Develop individualized recommendations for each centre, aimed at improving nursing practice, fostering teamwork, and consolidating NDM as a comprehensive primary care model.
6. Promote continuous improvement processes through the collective visualization of results and the automated proposal of strategic action plans, tailored to each PCT’s maturity level.

## 2. Materials and Methods

### 2.1. Study Design

This study employed a descriptive, cross-sectional, multicentre design underpinned by participatory and iterative methodologies, reflecting principles of community-based participatory research [16]. The primary aim was to evaluate the implementation and adoption of NDM across Catalan PCTs and identify factors influencing its consolidation. The project was conducted in three sequential phases:

1. Phase 1: Co-design of the digital evaluation tool (xGID) with stakeholders, through iterative feedback and refinement.
2. Phase 2: A small-scale implementation to test the draft questionnaire in practice, gather user feedback, and fine-tune the tool’s content and functionality.
3. Phase 3: Territorial-scale implementation to systematically assess NDM across multiple centres.

This paper presents the results of such territorial expansion.

### 2.2. Setting and Participants

The study was conducted across 47 PCTs in Catalonia, encompassing diverse geographic regions including urban, suburban, and semi-rural areas, primarily along the central and northern coast—although not exclusively. Centres participated voluntarily. Participants consisted of healthcare professionals from various disciplines: nurses, physicians, administrative staff, healthcare assistants, and other professional profiles. Individual’s eligibility required active employment at the participating centre and voluntary participation in on-site scheduled data collection sessions, resulting in a total sample of 1474 professionals.

### 2.3. Instrument: xGID Questionnaire

#### 2.3.1. Co-Design Process

A multidisciplinary working group of 9 experts (including frontline nurses, managers, and digital health specialists) participated in the co-design of the xGID questionnaire. Over iterative encounters and meetings from June to December 2022, iterative development cycles were employed, allowing continuous input and refinement [17,18]. During this phase, an initial version was piloted in five centres, where qualitative feedback informed revisions to item wording and platform usability. Subsequently, the revised instrument was tested in nine additional case studies to further validate its functionality. This participatory approach facilitated content validity, ensuring the tool accurately reflected frontline realities and strategic objectives of NDM.

#### 2.3.2. Structure and Content

The final questionnaire comprised 19 items grouped into three core domains (Table A1):

1. Relationship with the patient—the ability to adapt to patients’ needs, respect their autonomy, and apply a biopsychosocial approach.
2. Professional practice—reflects perceptions of professional autonomy, decision-making, and the use of guidelines and protocols.
3. Organizational conditions—encompasses continuity of care, accessibility, and teamwork in NDM practice.

These items were further categorized into seven dimensions to enable detailed diagnostic insights into NDM implementation (Table A2):

4. Understanding: The centre’s capacity to identify and interpret the nature and context of patients’ demands.
5. Managing: How decisions are made within the demand-management process.
6. Resolving: The effective degree to which demands are resolved.
7. Tools: Availability of material and digital resources.
8. Capacity: Structures and resources that support NDM.
9. Strategy: Strategic alignment of NDM with the centre’s overall plans.
10. Readiness: The willingness of the centre and the team to integrate NDM.

**Figure 1:**
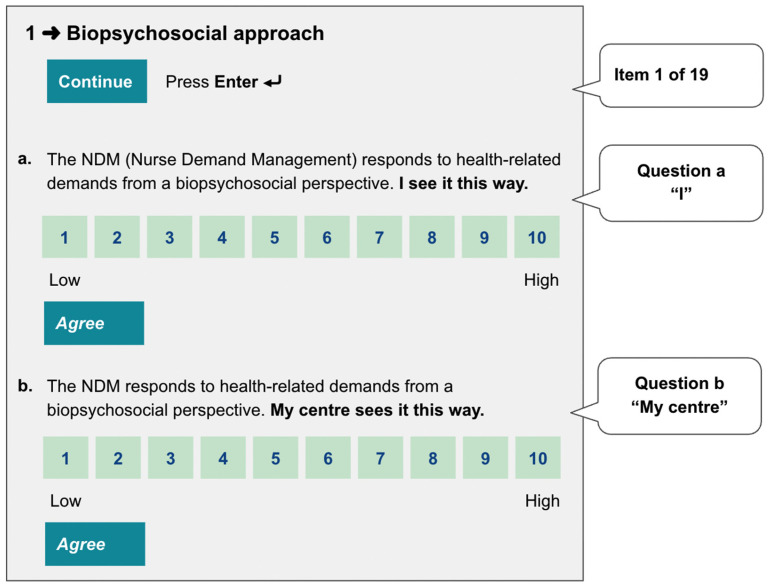
Preview of the double question structure as it is presented to the study participants by the evaluation tool.

Responses were structured through a ten-step dual-format Likert-type scale (1–10), capturing both individual professional self-assessment and their perception of collective practice at their centre (Figure 1). This dual approach aimed to mitigate the self-serving bias commonly encountered in professional self-assessments [19]. This double evaluation also aimed to reduce the difficulty participants might have in establishing what is the appropriate number in the scale to express their thoughts, which as a problem, as Sullivan pointed out when also defying such continuous Likert formats that are acceptable in psychometric research [20]. The ten-step scale was chosen in light of its frequent use in non-clinical evaluations and the potential ability to provide more nuanced scores when an “approved” grade could reasonably be given. More frequently used scales, such as those with 6 or 7 steps, have reduced the options to just 3 above the middle, potentially reducing all the answers to “5” on the 6-step scale and “5” and “6” on the 7-step scales.

### 2.4. Data Collection Procedure

**Figure 2:**
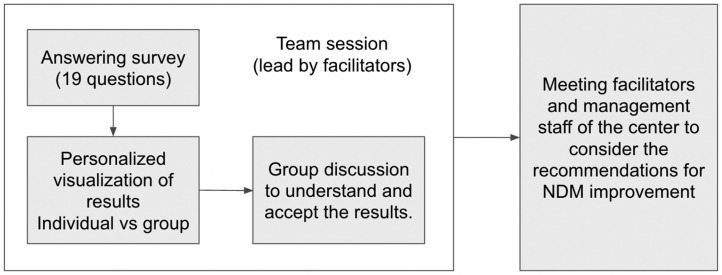
Workflow of the xGID evaluation.

Data collection occurred between January 2023 and October 2024 through facilitated, on-site sessions at each participating centre (Figure 2). These sessions were led by external nurse consultants who assisted participants in completing the online questionnaire using institutional devices or personal smartphones. To ensure anonymity and compliance with data protection regulations, the platform automatically assigned randomized, anonymous user codes. Demographic data were intentionally limited to professional role (nurse, physician, or administrative/other) and general age range.

### 2.5. Real-Time Feedback and Visual Analytics

**Figure 3:**
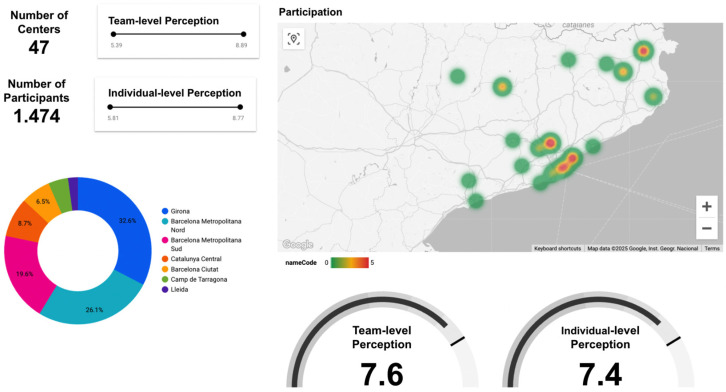
Centres comparison panel available to PCTs managers.

The xGID platform was designed to offer immediate, individualized feedback to respondents through visual dashboards comparing personal and collective scores. Aggregate team results were displayed to foster group discussion, reflection, and collective learning during the data collection sessions [21]. In Figure 2, the structure of the sessions is shown. Centre managers also had the ability to access additional comparative analytics across centres, aiming to facilitate strategic planning and action prioritization (Figure 3). This approach aligns with learning health system principles, enhancing continuous organizational improvement [22].

### 2.6. Questionnaire Development

All quantitative data were exported directly from the xGID platform via an app-enabled export service to enable data cleaning and analysis. No personally identifiable information was retained. The final dataset included an anonymous user ID, an anonymous centre ID, the associated healthcare provider, the health region of Catalonia, the centre’s reference population (for contextual purposes), responses to each questionnaire item, and the respondent’s professional profile. Incomplete or partial responses were excluded to ensure analytic consistency.

The analysis focused on three perspectives: (1) single-item performance by centre, (2) aggregated (composite) scores by centre, and (3) single-item responses by professional profile. For each, standard descriptive statistics were computed: count, mean, median, first quartile (Q1), third quartile (Q3), interquartile range (IQR), minimum, maximum, and standard deviation (SD). For centre-level analyses (perspectives 1 and 2), results were subsequently aggregated at the system level by calculating descriptive statistics over the distribution of centre-level means. This approach ensures equal weighting of centres, avoiding bias from differing response volumes.

Composite indicators were calculated per respondent using the weighting and clustering schemes detailed in Appendix A Table A1 and Table A2. These composite scores, as well as single-question results, were only analyzed for centres with sufficient response volume. A general NDM-implementation index—automatically generated by the xGID platform—was excluded from this article due to its pedagogical rather than analytical purpose.

Analyses based on professional profile followed the same descriptive methodology, with filtering based on professional roles instead of centres. This aimed to identify patterns potentially linked to system-wide policies or regulations beyond individual centre dynamics.

Although Likert-scale data are ordinal, the 10-point format approximates interval properties when distributional assumptions are not violated. Mean scores were therefore used for ease of comparison and aggregation, alongside medians and IQRs to account for potential skewness [20].

### 2.7. Statistical Analysis

For the answers pertaining to the centre perception, multiple statistical analysis have been conducted. The Kruskal–Wallis Test and Dunn post hoc analysis have been performed for questions and composite metrics to identify and parametrize significant differences between professional profiles and identify potential bias in the final results due to the mix of professionals and their respective perceptions and experiences. Kruskal–Wallis analysis has been used to identify questions with significant differences among profiles (*p* < 0.05) and in such cases, we conducted Dunn’s pairwise comparisons with Bonferroni correction. This allowed us to specifically identify which pairs of groups (nurse vs. admin; nurse vs. physician) differed significantly for each item. The same analysis was performed for the ten domains/dimensions (composite metrics).

To identify to what degree obtained scores can be attributed to differences between the centres or between professionals (for centre responses), the variance between centres’ means and within-centre responses (i.e., individual responses for a centre) for each question and composite metric was calculated. With the two variances, the intraclass correlation was subsequently calculated.

## 3. Results

### 3.1. Participation

The NDM evaluation tool was implemented in a total of 47 PCTs across Catalonia, collecting data from 1474 professionals (see full data in Table S1). The average response rate per centre was 58.4%, ranging from a minimum of 32.8% to a maximum of 86.2%. This level of participation ensures an overall significant and representative sample for evaluating NDM in most centres and in all regions.

During the pilot phase, age data were found to be potentially identifying in centres with small staff size; as a result, such information was excluded from statistical reporting and permanently removed from records to uphold confidentiality.

### 3.2. Global Results

**Figure 4:**
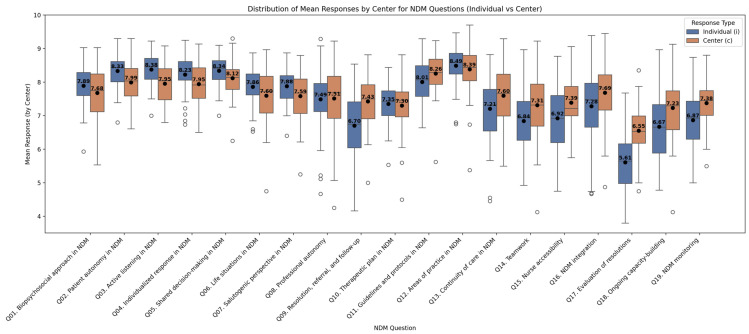
Comparison of individual self-assessment score (blue) and centre self-assessment score (orange). Dots indicate the mean score, while bars indicate the median score, circles are the scores of outlayer centers.

The global average for all participating centres for NDM implementation was 7.56 points out of 10 on the 1–10 scale, with individual centres’ scores ranging from 6.8 to 8.5. The professionals’ self-assessment (“I”) was slightly lower at 7.43. This pattern—being more self-critical on an individual level and more positive on a collective level—was consistently observed for most questions across all results (see Figure 4).

**Figure 5:**
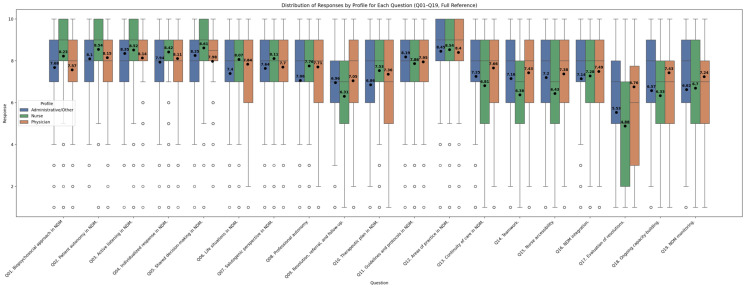
Comparison of centre self-assessment score by professional groups. Dots indicate the mean score, while bars indicate the median score, circles are outlying scores by individuals.

Among participating professionals, differences are also observable between profiles. Nurses tend to provide more stringent ratings, especially regarding organizational issues and collective decision-making. On the other side, physicians show slightly higher ratings, reflecting a generally more positive view of the centre’s functioning regarding NDM. Administrative staff and other professionals offer overall balanced scores with lower dispersion, and they rate accessibility and continuity particularly highly. These differences can be clearly observed in Figure 5, which plots the arithmetic means, median and IQR by item according to professional profile.

**Figure 6:**
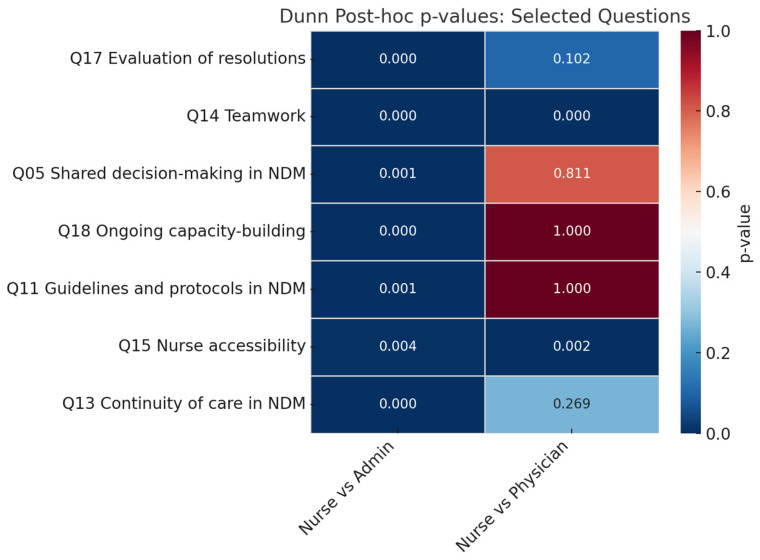
Dunn analysis between professional profiles coloured in a heat map for questions with a *p* < 0.05 in Kruskal–Wallis Test. Intense blue colours indicate high significance (*p* < 0.05) in the variability of answers between profiles.

The Kruskal–Wallis test and Dunn post hoc analysis (Figure 6 for the questions with lower ICC scores, see Table S2 for ICC and Table S3 for Kruskal-Wallis analysis) are consistent with what we can observe in Figure 5. They further highlight a greater difference between admins and nurses than that between physicians and nurses. While the first two have differing perceptions (*p* < 0.05) of guidelines and protocols, care continuity, teamwork, accessibility, and evaluation of resolutions and ongoing capacity building, physicians and nurses only differed in their perceptions of teamwork and accessibility, and to some degree in active listening (*p* = 0.095) and evaluation of resolutions (*p* = 0.102).

### 3.3. Results by Dimension

**Figure 7:**
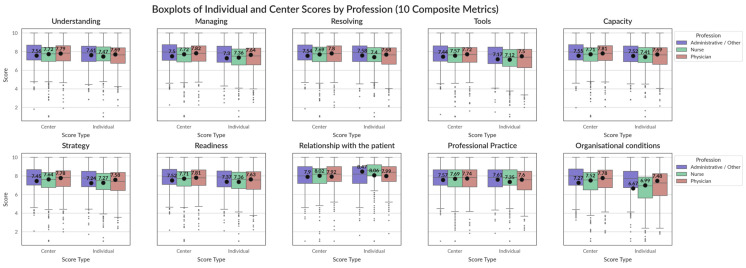
Comparison means of centre responses for each composite dimension (weighted aggregate of the responses of multiple questions following Table A1 and Table A2) for the different professional profiles. Dots indicate the mean score, while bars indicate the median score, circles are outlaying scores.

**Figure 8:**
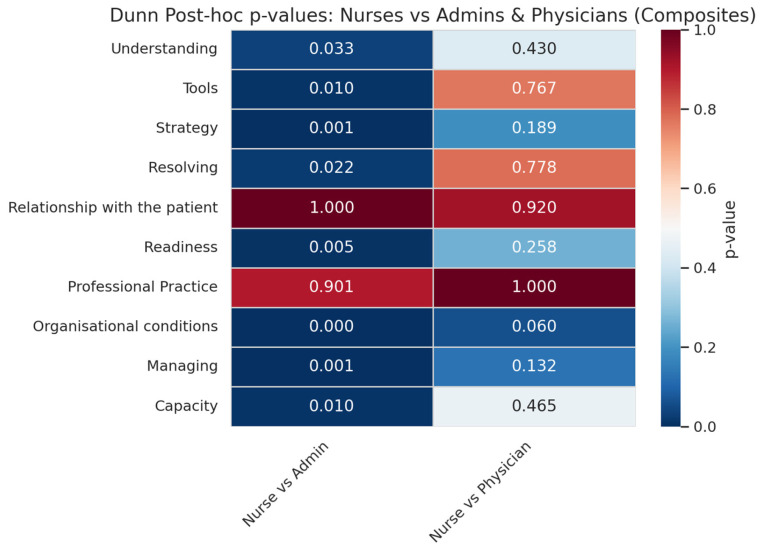
Dunn analysis between professional profiles coloured in a heat map for all dimensions. Intense blue colours indicate high significance (*p* < 0.05) in the variability of answers between profiles.

Dimensions show a general overview of centres’ performances in general areas of intervention (Figure 7). The results show that areas such as the “organizational conditions” or the availability of tools and strategy are underperforming other metrics. This is especially true for “organizational conditions”, which see generally low scores on the individual level as well. Meanwhile, the “relationship with the patient” is the exception to all the metrics, where individual performance is regarded as superior to that of the centre, although even the centre’s performance is superior to all the other metrics (a mean of 7.96 for “relationship with the patient”, followed by a mean of 7.66 for “understanding”). When it comes to significance of differences between professional profiles (Figure 8) we see again the nurses and professionals have a greater level of shared experiences except for “strategy”, “managing” and “organizational conditions” although none of them crosses the significance threshold.

### 3.4. Differences Between Centres and Individuals in the Determination of Results

To determine if differences in scores were due to the functioning of specific centres (differences in scores between centres) or a generic situation experienced across centres (differences in scores within centres), the intraclass correlation coefficient (ICC) was calculated for questions and composite metrics. For the 19 individual items, the median ICC fell to 0.093 (range 0.043–0.158), showing that item-level perceptions are more heterogeneous within centres than across them (Table 1).

For the 10 composite dimensions, the median ICC was 0.165 (range 0.122–0.179), indicating that ≈17% of total variance is attributable to differences between centres (Table 2).

## 4. Discussion

### 4.1. Interpretation of Findings

Analysis of the 1474 survey responses from 47 primary care teams (PCTs) reveals three complementary insights that speak directly to the challenges framed in the introduction.

#### 4.1.1. A Frontline Perception Gap Between Nurses and Other Professionals

Kruskal–Wallis tests revealed statistically significant differences by professional group in 15 of the 29 questionnaire items (52%), with the largest gaps in “teamwork” (χ^2^, *p* < 0.0001) and “organizational conditions” (*p* < 0.001). These disparities were especially pronounced between nurses and administrative staff, with Dunn tests showing consistent differences across most domains except “relationship with the patient” and “professional practice”. Differences with physicians were more nuanced but notable in domains such as “readiness” and “organizational conditions” (*p* = 0.258 and 0.060, respectively). This suggests that nurses, as frontline professionals, are more attuned to the disconnect between the care they aspire to deliver and the resources available to them.

These findings echo existing evidence that autonomy is an experienced, not merely assigned, condition. Those tasked with implementing NDM perceive constraints more acutely [23], whereas physicians tend to offer more uniformly positive assessments, likely reflecting a more structural or system-level viewpoint. This divergence is not indicative of conflict but of shared, yet unresolved, challenges. Notably, perceptions of “Teamwork” differ substantially: physicians may either assume nurses are collaborating or question whether collaboration is genuinely occurring. Addressing this perception gap presents a key opportunity for targeted professional development and resourcing strategies. Providing nurses with appropriate tools and capacity could enhance their perception of support and strengthen centre-wide implementation.

#### 4.1.2. Marked Heterogeneity in “Professional Practice” and “Organizational Conditions” Domains

The gap between the highest- and lowest-performing centres reached 4.04 points in the “professional practice” domain and 3.91 points in “organizational conditions”, compared to 2.44 points in “relationship with the patient”. Intraclass correlation coefficients (ICCs) averaged 0.12 across all items (range: 0.04–0.18) and rose to 0.15 for “professional practice”, indicating that approximately one-eighth of the variance is attributable to centre affiliation—generally the threshold for statistical significance in ICCs.

Most composite scores exceeded an ICC of 0.15, suggesting that while responses to individual items varied substantially within centres, overall domain scores were more centre-dependent. This underscores that NDM implementation is currently highly contingent on the individual centre’s context and leadership. The lack of consistent system-wide support suggests that responsibility for implementation rests largely with centre management. These findings reinforce the study’s conclusion that implementation maturity is uneven and highlight the need for policies that promote cross-centre alignment in NDM practices.

#### 4.1.3. A Self-Critical Bias That Can Fuel Collective Learning

Participants rated their own practice slightly lower than that of their team (mean 7.43 vs. 7.56), a consistent difference across most items (Figure 4), except for the initial questions. Rather than indicating complacency, this self-critical perspective suggests a latent readiness for reflective practice—an avenue that is worthy of further exploration to deepen our understanding of professionals’ experiences within their centres. This finding, though unintended, emerged from the study’s design, which did not collect data to fully explain these discrepancies. Notably, the paired question format was initially intended to account for self-serving bias. As a result, the underlying causes of this lower self-assessment remain unexplored, underscoring the need for complementary qualitative methods such as interviews, focus groups, journaling, or shadowing.

Beyond these general patterns, the discrepancy is more pronounced in items related to “organizational conditions”, where participants rated their organizations more favourably than their own experiences. For instance, items on “teamwork” and “capacity-building” fall into this category. While no clear explanation is evident for the teamwork dimension, the capacity-building gap may reflect perceived inequities—particularly frustration among nurses and administrative staff that physicians receive more training opportunities. This interpretation is supported by Figure 6 and aligns with prior research indicating that some younger nurses feel constrained in their clinical autonomy due to legal and training limitations [24]. Nonetheless, the findings are inconclusive, as both “organizational conditions” and “capacity-building” dimensions yielded modest intraclass correlation coefficients (ICC = 0.145 and 0.111, respectively).

### 4.2. Policy Implications and Targeted Recommendations

The current healthcare and social reality is characterized by increasing clinical and social complexity, alongside a marked rise in health needs and demands—particularly among the elderly, the dependent, and those living alone. It is generally accepted that nurses hold great potential in delivering more effective and efficient primary healthcare, and it is based upon this idea that NDM is implemented. Yet, its impact is diluted by uneven organizational backing and lingering professional silos, as we have demonstrated. To convert promising practice into system-wide value, certain interventions are necessary.

The granular performance signals generated by xGID enable a policy response that is both stratified and data-driven. The authors identify three clusters of domains in which distinctive action is needed: (i) uniformly weak domains (low mean ≤ 7.5; low ICC), (ii) strong but heterogeneous domains (high mean > 7.5; high ICC ≥ 0.15), and (iii) strong and consistent domains (high mean; low ICC). Each profile demands a different policy change, summarized below.

#### 4.2.1. System-Wide Recommendations

Domains with low mean scores and low intraclass correlation coefficients (ICCs)—for example, evaluation of resolutions (=6.56), ongoing capacity-building (7.25) and teamwork (7.34)—reflect system-wide under-investment rather than centre-specific failure. Because these weaknesses jeopardize NDM’s promise of resolving multiple problems in a single encounter and preventing bureaucratic “ping-pong”, five mutually reinforcing measures are recommended:

- Include NDM results within the national health data dashboard (e.g., Central de Resultats in Catalonia).
- Offer adequate educational and formative opportunities for the tasks and abilities which fall under NDM and for which nurses can take more autonomous roles without requiring a physician, including equipping nurses to manage the growing clinical and social complexity of an ageing population.
- Same-day tele-slots and community outreach clinics operationalise the NDM principle of patients seeing the “most appropriate professional, first time,” relieving pressure on physicians while improving continuity for vulnerable groups who otherwise default to emergency services.

Additionally, reviewing the definition of NDM and what it implies is also a necessary course of action that should be taken at a systemic level, as differences might occur on a healthcare provider basis due to different interpretations of NDM.

#### 4.2.2. Enable Equality Among Centers

High-mean, high-variance domains—such as Readiness, Strategy, Capacity, Professional Practice—display excellence in some centres and inertia in others (ICC ≥ 0.15). Fragmented implementation magnifies inequity and inflates avoidable appointments; translational policy should emphasize diffusion through the universal availability of staffing models, triage algorithms, and digital tools, accelerating horizontal learning while reducing the cognitive load generated by reinventing workflows locally.

#### 4.2.3. Well-Performing Areas

Person-centred competencies such as Areas of Practice (8.39) and Shared Decision-Making (8.12) already perform at scale with minimal variance, signalling a nascent standard of care that can safely be codified and redistributed:

- Embedding these high-performing competencies at undergraduate and postgraduate levels will secure early socialization into evidence-based, person-centred practice [25].
- Enabling communities of practice and learning for nurses will allow the spread of tacit skills across settings, an approach congruent with the salutogenic framing of NDM and its emphasis on patient autonomy.

#### 4.2.4. Centre-Specific Recommendations

While the recommendations primarily address global policy and capacity, centre-level action is also essential. Variability across centres—some excelling in NDM implementation while others lag behind—highlights the importance of local engagement. National efforts risk being ineffective if professionals and centre managers do not act on them. To support this, the xGID platform not only collected data but also provided each centre with a general assessment. The content of these messages is available in the Supplementary Materials (see Table S4).

### 4.3. Methodological Strengths and Limitations

#### 4.3.1. Strengths and Contributions to Nurses’ Autonomy Evaluation

The high—but uneven—uptake observed aligns with international evidence showing that nurse-led demand management (NDM) and other advanced practices thrive only where organizational readiness, enabling legal frameworks, and supportive local leadership intersect [23,26,27]. This supports our conceptual framing: professional autonomy is a negotiated, context-sensitive capability, not a fixed entitlement.

At the regional level, prior single- and multicentre studies in Catalonia have reported resolution rates of 50.9% to 62.5% for minor acute conditions [9,28], alongside high acceptance of nurse-led care by both patients and professionals. However, these studies offered limited insight into the organizational heterogeneity influencing implementation depth.

This study contributes in two key ways. First, it operationalises implementation maturity through practitioner-level collective intelligence metrics rather than relying on proxy indicators like guideline adoption. Second, it shows how these metrics can be fed back to teams in near real-time, transforming measurement into a tool for continuous improvement. Together, these findings support our central claim: maturity in NDM cannot be inferred from adoption alone—it must be evaluated using practitioner-centred, context-aware intelligence.

Key strengths include the participatory co-design of the instrument, a large and geographically diverse sample (47 PCTs; *n* = 1474), and immediate visual feedback that facilitated open discussion of NDM experiences. The xGID system identified both consolidated strengths—such as professional autonomy and scope of practice—and critical gaps, including teamwork, longitudinal care continuity, and ongoing professional development. The latter is particularly pressing given prior findings that younger nurses perceive constraints on their autonomy due to legal and training limitations [24].

Notably, the lowest and most polarized scores were linked to organizational design elements—such as accessibility and monitoring—indicating systemic shortcomings. Beyond diagnosis, xGID functions as a catalyst for collective intelligence processes, consistent with the framework proposed by Monguet et al. [21] in the context of healthcare consensus-building. By combining metrics, shared visualizations, and structured discussion, the system fosters organizational learning and strategic alignment.

This participatory approach is especially valuable in complex environments where implementing innovations like NDM requires coordination across diverse stakeholders. However, our findings point towards the continued need to build shared understanding around NDM, as diverging perspectives persist due to differing expectations and interpretations of nurses’ roles [29].

#### 4.3.2. Limitations of the Present Research

The voluntary enrolment of centres introduces the risk of selection bias towards more innovation-oriented teams. Nonetheless the authors deem this risk to be marginal given the large amount of predisposition of centres to participate in the study, even from those centres for which the researchers expected low NDM performance. The cross-sectional design precludes causal inference, and the reliance on self-reported Likert data may be subject to common-method bias. For example, a Likert scale might fail in addressing a predisposition to “approve” the centre or themselves and submit qualifications above “5”. For more accurate research on the barriers to NDM implementation, other sources of (qualitative) data should complement the research. In fact, as data-gathering sessions were performed on-site and discussions were held with the professionals, these sessions remain an untapped source of valuable qualitative data which could help further substantiate the findings of this article and dive deeper into the origins of the scores. For example, during some sessions, questions were brought up about what NDM was by all sorts of professional profiles who had a limited knowledge of the programme they were supposed to implement. This adds to not incorporating in-depth and structured qualitative data or interviews with centre managers during this research, limiting the understanding of contextual factors unique to each setting.

Another shortcoming of the study is the management of the centre and individual scores, as the original design intended to use the centre scores as a more balanced form after asking for the personal performance. Yet, the lack of self-serving bias poses additional questions to the suitability of this double-question approach and might require a revaluation of how the data from the two questions should be managed. Finally, the study captures at this phase, just a part of the whole Catalan health service in primary care, more centres need to be evaluated to have a more complete picture, which will still be static in time. Because NDM implementation in ongoing and new measures are taken these results might change through time.

#### 4.3.3. Future Research

Longitudinal follow-up using xGID will allow us to track whether action plans to improve NDM implementation translate into measurable improvements. Such follow-up should comprise a greater amount of primary care centres of Catalonia in order to provide a greater representativeness of the Catalan healthcare system. Given the ease of use of the evaluation on a standalone platform that centres can use quasi-autonomously, yearly evaluations can be performed and compared, on a centre basis and system-wide. Qualitative work—particularly ethnographic observations and semi-structured interviews (potentially leveraging the evaluation sessions performed in centres)—should explore the micro-processes that shape professional autonomy, interprofessional negotiation, and patient experience. Nonetheless, due to the large amount of centres within the region, results from the xGID survey could be used to choose a limited representative sample of centres with differing characteristics for the qualitative methods.

## 5. Conclusions

Through this study, it is made evident that although NDM is implemented in the studied regions of the Catalan Health System, it is not harmoniously performed. In fact, disparities of up to 4 points in the scores of professional practice category among centres demonstrate that while some centres seem to perform well, others fall substantially behind. Such a statement is further substantiated by the high deviation between centres regarding professionals’ autonomy and capacity to intervene in therapeutic plans. Similarly, there are also a lack of organizational conditions and tools available to effectively operate and implement the NDM, especially evaluation and monitoring systems. Overall, through this evaluation approach, we identify notable gaps in the implementation of the NDM, providing a clearer picture of how its implementation is uneven and where additional efforts should be made by authorities to fulfil the promise of an improved healthcare service through the implementation of NDM.

## Figures and Tables

**Table 1:** Intraclass correlation coefficient for each of the questions. Those >0.15 are considered significant at an organization level (i.e., the centre makes a difference in the experience of NDM implementation), while those <0.05 are considered only significant at an individual level (i.e., being in one centre or another is irrelevant to the NDM experience).

Item	ICC
Q01. Biopsychosocial approach in NDM.	0.116
Q02. Patient autonomy in NDM.	0.093
Q03. Active listening in NDM.	0.086
Q04. Individualized response in NDM.	0.092
Q05. Shared decision-making in NDM.	0.062
Q06. Life situations in NDM.	0.113
Q07. Salutogenic perspective in NDM.	0.109
Q08. Professional autonomy.	0.158
Q09. Resolution, referral, and follow-up.	0.100
Q10. Therapeutic plan in NDM.	0.083
Q11. Guidelines and protocols in NDM.	0.050
Q12. Areas of practice in NDM.	0.076
Q13. Continuity of care in NDM.	0.100
Q14. Teamwork.	0.119
Q15. Nurse accessibility.	0.079
Q16. NDM integration.	0.146
Q17. Evaluation of resolutions.	0.043
Q18. Ongoing capacity-building.	0.111
Q19. NDM monitoring.	0.077

**Table 2:** Intraclass correlation coefficient for each of the dimensions (composite metrics). Those >0.15 are considered significant at an organization level (i.e., the centre makes a difference in the experience of NDM implementation), while those <0.05 are considered only significant at an individual level (i.e., being in one centre or another is irrelevant for the NDM experience).

Item	ICC
Understanding	0.166
Managing	0.163
Resolving	0.167
Tools	0.156
Capacity	0.167
Strategy	0.173
Readiness	0.179
Relationship with the patient	0.122
Professional Practice	0.154
Organizational conditions	0.145

## Data Availability

The datasets presented in this article are made available in the Supplementary Materials of the present article.

## Supplementary Materials

The following supporting information can be downloaded at https://www.mdpi.com/article/10.3390/healthcare13121403/s1, Table S1: Dataset; Table S2: icc_df; Table S3: kurskal_df; Table S4: Recommendations.

## Abbreviations

The following abbreviations are used in this manuscript:

NDM	Nurse Demand Management.
PCT	Primary Care Team.
GID	NDM (in Catalan).

## Appendix A

**Table A1:** The 19 questions used in the study to evaluate NDM implementation.

Item	Question
Block 1. Relationship with the patient.	
Q01. Biopsychosocial approach in NDM.	Nurses in my PCT address patients’ health demands from a biopsychosocial standpoint.
Q02. Patient autonomy in NDM.	Nurses in my PCT take into account the autonomy and responsibility of the patient.
Q03. Active listening in NDM.	Nurses in my PCT practice active listening, considering family and community contexts.
Q04. Individualized response in NDM.	Nurses in my PCT respond to the unique needs of each patient.
Q05. Shared decision-making in NDM.	Nurses in my PCT respect the patient’s decisions and preferences.
Block 2. Professional Practice	
Q06. Life situations in NDM.	Nurses in my PCT analyse and assess problems and/or life situations to issue a diagnosis.
Q07. Salutogenic perspective in NDM.	Nurses in my PCT prioritize interventions from a salutogenic perspective.
Q08. Professional autonomy.	Nurses in my PCT exercise NDM with professional autonomy.
Q09. Resolution, referral, and follow-up.	Within NDM, nurses in my PCT have all necessary resources to provide effective solutions.
Q10. Therapeutic plan in NDM.	Nurses in my PCT propose personalized therapeutic plans.
Q11. Guidelines and protocols in NDM.	Decision-making is aided by best-practice guidelines and protocols.
Block 3. Organizational conditions	
Q12. Areas of practice in NDM.	In my PCT, NDM services are provided in person (at the centre, home, school, or nursing home), by phone, or virtually.
Q13. Continuity of care in NDM.	My PCT is organized so that each nurse cares for the same assigned population over time.
Q14. Teamwork.	NDM in my PCT maximizes teamwork.
Q15. Nurse accessibility.	NDM in my PCT improves patients’ access to their primary nurse.
Q16. NDM integration.	NDM is well-integrated in my PCT.
Q17. Evaluation of resolutions.	EQA indicators effectively measure the resolution level of NDM.
Q18. Ongoing capacity-building.	Within my PCT, and in the context of NDM, skills and knowledge are continually updated.
Q19. NDM monitoring.	The organization monitors and reviews NDM indicators.

**Table A2:** Ponderation of each question for the evaluation indicators. All scores are always provided on a scale of 1–10, including after pondering results.

Item	Understanding	Managing	Resolving	Tools	Capacity	Strategy	Readiness
Q01. Biopsychosocial approach in NDM.	4	1	2	2	2	4	2
Q02. Patient autonomy in NDM.	2	1	4	1	4	2	2
Q03. Active listening in NDM.	4	4	1		4	1	2
Q04. Individualized response in NDM.	2	2	4	1	2	2	2
Q05. Shared decision-making in NDM.	2	4	1	1	4	1	2
Q06. Life situations in NDM.	4	2	4	2	4	2	1
Q07. Salutogenic perspective in NDM.	4	2	2	1	4	2	2
Q08. Professional autonomy.	2	4	4	2	4	2	4
Q09. Resolution, referral, and follow-up.	2	2	4	2	4	2	4
Q10. Therapeutic plan in NDM.	2	1	4	4	2	2	2
Q11. Guidelines and protocols in NDM.	2	4	2	4	2	2	1
Q12. Areas of practice in NDM.	2	4	2	1	4	2	4
Q13. Continuity of care in NDM.	2	4	2	0	2	4	2
Q14. Teamwork.	1	4	2	1	2	4	4
Q15. Nurse accessibility.	2	4	0	1	0	4	2
Q16. NDM integration.	1	2	0	4	1	4	4
Q17. Evaluation of resolutions.	2	4	2	4	4	2	2
Q18. Ongoing capacity-building.	2	4	1	2	4	4	2
Q19. NDM monitoring.	2	4	1	4	2	4	2
